# Deep Federated Adaptation: An Adaptative Residential Load Forecasting Approach with Federated Learning

**DOI:** 10.3390/s22093264

**Published:** 2022-04-24

**Authors:** Yuan Shi, Xianze Xu

**Affiliations:** Electronic Information School, Wuhan University, Wuhan 430072, China; shiyuan@whu.edu.cn

**Keywords:** electric load forecasting, transfer learning, federated learning, domain adaptation

## Abstract

Residential-level short-term load forecasting (STLF) is significant for power system operation. Data-driven forecasting models, especially machine-learning-based models, are sensitive to the amount of data. However, privacy and security concerns raised by supervision departments and users limit the data for sharing. Meanwhile, the limited data from the newly built houses are not sufficient to support building a powerful model. Another problem is that the data from different houses are in a non-identical and independent distribution (non-IID), which makes the general model fail in predicting accurate load for the specific house. Even though we can build a model corresponding to each house, it costs a large computation time. We first propose a federated transfer learning approach applied in STLF, deep federated adaptation (DFA), to deal with the aforementioned problems. This approach adopts the federated learning architecture to train a global model without undermining privacy, and then the model leverage multiple kernel variant of maximum mean discrepancies (MK-MMD) to fine-tune the global model, which makes the model adapted to the specific house’s prediction task. Experimental results on the real residential datasets show that DFA has the best forecasting performance compared with other baseline models and the federated architecture of DFA has a remarkable superiority in computation time. The framework of DFA is extended with alternative transfer learning methods and all of them achieve good performances on STLF.

## 1. Introduction

The International Energy Agency has identified energy efficiency in buildings as one of the five methods to secure long-term decarbonization of the energy sector [1]. In addition to environmental benefits, the improvement of the building energy efficiency also presents vast economic benefits. Buildings with efficient energy systems and management strategies have much lower operating costs [2]. The activities of humans in residences occupy a large portion of energy consumption and CO_2_ emission [3]. Residential load forecasting can assist sectors in balancing the generation and consumption of electricity, which improves energy efficiency through the management and conservation of energy.

Several uncertain factors, such as historical load records, weather conditions, population mobilities, social factors and emergencies, influence electricity usage. Due to the high volatility and uncertainties involved, short-term load forecasting for a single residential unit may be more challenging than for an industrial building [4]. Machine-learning-based methods, driven by data, are applied to mitigate these challenges more and more frequently. However, the scope of machine-learning-based applications will be hindered due to the privacy and security concerns raised by more and more supervision departments and users. Even in some countries, many users refuse the installation of smart meters because users are reluctant to disclose their private data. In addition, newly built houses cannot provide sufficient data to build effective models. In summary, the data exist in the form of isolated islands, which makes it difficult to merge the data from different users to train a robust model. Hence, one of the problems in this paper we focused on is data availability and privacy.

A number of researches have achieved good results on STLF, such as support vector regression (SVR) [5], the artificial neural network [6] and boosted tree [7]. Additionally, some hybrid methods that combine artificial intelligence methods with traditional methods are proposed to achieve better forecasting performance, such as hybridizing extended Kalman Filter and ELM [8]. Fan et al. [9] proposed a SVR model hybridized with differential empirical mode decomposition (DEMD) method and auto regression (AR) for electric load forecasting. Transformer is a novel time series prediction model based on the encoder–decoder structure. Originating from this structure, many methods have yielded good results in the field of energy forecasting, such as STA-AED [10] and informer [11]. However, these approaches do not consider user privacy and modeling with limited data.

A lot of privacy-preserving solutions relying on data aggregation and obfuscation have been proposed to ensure privacy [12]. However, these solutions are not suitable for residential short-term energy forecasting since they often introduce extra procedures to obfuscate and reconstruct the data [13]. In addition, as the solutions based on machine learning are computationally intensive in the step of model training, most works consider only centralized training approaches. Clients’ data should be collected onto a central server where the model is trained, which leads to a heavy burden on communication. Especially when the model needs to be constantly updated with new data, as the data from millions of distributed meters are required. Under this circumstance, federated learning has been proposed to overcome these challenges. Federated Learning is a distributed machine learning approach where a shared global model is trained, under the coordination of a central entity, by a federation of participating devices [14]. The peculiarity of the approach is that each device trains a local model with the data never leaving each local machine. Only the parameters of models are sent to the central computing server for updating the shared global model. Hence, the federated architecture can protect privacy effectively. Federated learning has been demonstrated to be effective in the area of load forecasting, federated learning with clustered aggregation is proposed in [15], and has good performance for individual load forecasting. Federated learning applied in heating load demand prediction of buildings also has a high capability of producing produce acceptable forecasts while preserving data privacy and eliminating the dependence of the model on the training data [16]. Furthermore, federated learning has been applied in several application successfully, such as human–computer interaction [17], natural language processing [18], healthcare classification [19], transportation [20,21], and so on, where privacy and scalability are essential.

Another critical problem for residential load forecasting is that the general model is not adapted to each house since the datasets are non-IID, which the federated architecture and conventional machine learning algorithms do not well handle with [22]. The problem is particularly acute in the case of newly built houses. Even though the dataset bias and unbalance are inevitable [23], many researchers classify users according to different attributes to deal with this challenge, but it does not fit well with a federated learning architecture [24]. This situation is particularly suitable for applying transfer learning. Transfer learning aims at establishing knowledge transfer to bridge different domains of substantial distribution discrepancies. In other words, data from different houses have domain discrepancies which is a major obstacle in adapting the predictive model across users. STLF models based on transfer learning are discussed in [4,25,26].

A representative transfer learning method is domain adaptation, which can leverage the data in the information-rich source domain to enhance the performance of the model in the data-limited target domain. As a well-known algorithm applied for domain adaptation, deep neural network [27] is capable of discovering factors of variations underlying the houses’ historical data, and group features hierarchically in accordance with their relatedness to invariant factors, and it has been studied extensively. A lot of research has shown that deep neural networks can learn more transferable features for domain adaptation [28]. It is shown that deep features must eventually transition from general to specific in the network, with the transferability of features decreasing significantly at higher levels as domain discrepancies increase. In other words, the common features between different users are captured in lower layers, and the features of the specific user hide in higher layers which depend greatly on the target datasets and are not safely transferable to another user.

In this article, we address the aforementioned challenges within a novel user adaptative load forecasting approach. The approach is the combination of federated learning and transfer learning. The architecture of federated learning in this approach aims at building a CNN-LSTM based general model, which does not compromise privacy and works well with the limited data. Then, MK-MMD, a distance to measure domain discrepancies, is used to calculate the domain discrepancies between houses, then optimize the general network which can reduce the domain discrepancies effectively and reduce the forecasting error. The contributions of this paper are summarized as follows:

1. We propose a novel federated transfer approach DFA for residential STLF, which adopts a federated architecture to address the problems of data availability and privacy, and leverages transfer learning to deal with the non-IID datasets for improving forecasting performance;
2. DFA is investigated for STLF of residential houses and has shown remarkable advantages in forecasting performance over other baseline models. Especially, the federated architecture is superior to the centralized architecture in computation time;
3. The framework of DFA is extended with alternative transfer learning methods and all of them achieve good performances on STLF.

## 2. Technical Background

### 2.1. Federated Learning Concepts

Due to security and privacy concerns, data exist in the form of isolated islands, making it difficult for data-driven models to leverage big data. One possible approach is federated learning, which can train a machine learning model in a distributed way.

Let matrix $D_{i}$ denote the data held by the partner *i*, each row of the matrix represents one sample, and each column is a feature. Since the feature and sample spaces of the data parties may not be identical, federated learning can be classified into three classes: horizontally federated learning, vertically federated learning and federated transfer learning.

Horizontal federated learning is applicable in the conditions in which different partners have the same or overlapped feature spaces but different spaces in samples. It is similar to the case of dividing data horizontally in a tabular view, hence horizontal federated learning is also known as sample-partitioned federated learning. Horizontal federated learning can be summarized as Formula (1):

(1) $$X_{i}=X_{j},Y_{i}=Y_{j},I_{i}\neq I_{j},\forall D_{i},D_{j},i\neq j$$

let X, Y, I denote the feature space, the label space and the sample ID space.

Different from horizontal federated learning, partners in the vertically federated learning share the same spaces in samples, but different ones in feature spaces. We can summarize vertically federated learning as shown in Formula (2):

(2) $$X_{i}\neq X_{j},Y_{i}\neq Y_{j},I_{i}=I_{j},\forall D_{i},D_{j},i\neq j$$

Federated transfer learning is applied in the conditions in which datasets differ not only in sample spaces but also in feature spaces. For example, a common representation or model is learned from different feature spaces and later used to make predictions for samples with only one-side features. Federated transfer learning is summarized as shown in Formula (3):

(3) $$X_{i}\neq X_{j},Y_{i}\neq Y_{j},I_{i}\neq I_{j},\forall D_{i},D_{j},i\neq j$$

**Figure 1:**
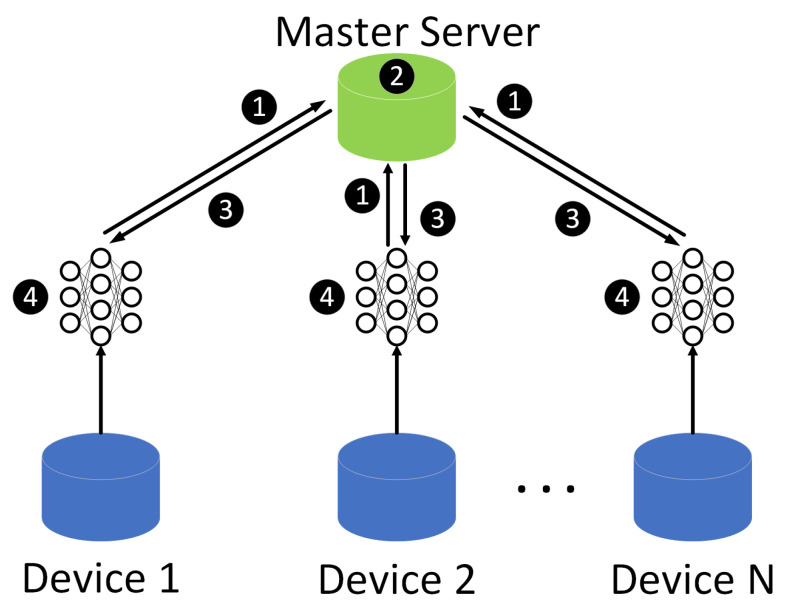
A horizontal federated learning architecture.

In this paper, the federated learning framework is a horizontal federated learning architecture since the data collected by devices is in the same feature space. It uses a master–slave architecture, as shown in Figure 1. In this system, N participant devices collaborate to train a machine learning model with the help of the master server.

In step 1, each participant computes the model gradient locally and masks the gradient information using cryptographic techniques such as homomorphic encryption, and sends the results to the master server. In step 2, the master server performs a secure aggregation operation. In step 3, the server distributes the aggregated results to each participant. In step 4, each participant decrypts the received gradients and updates their respective model parameters using the decrypted gradients. The above steps continue iteratively until the loss function converges or the maximum number of iterations is reached. We can see that the data of the participants are not moved during the training process, so the federated learning can protect user privacy that distributed machine learning models trained on Hadoop do not have. In the training process, an arbitrary number of devices can concur to model training without the need of transferring collected data to a centralized location. The federated model can tackle the increasing data without consideration of communication bandwidth since only local gradients need to be sent.

### 2.2. Transfer Learning Concepts and MK-MMD

Firstly, it is hard to collect sufficient data from domains of interest, referred to as target domains. Meanwhile, a large number of data may be available for some related domains called source domains. Secondly, machine learning algorithms work well based on a fundamental assumption: the training and future data must be in the same feature space and follow the same distribution. However, this assumption is not held in real-world applications. For these reasons, transfer learning is introduced to address these problems. Transfer learning can leverage similarities between data, tasks, or models to conduct knowledge transfer from the source domain to the target domain. These similarities are considered a representation of the distance between domains. Then the key issue is to introduce the standard distribution distance metric and minimize the distance.

MK-MMD is a type of distance metrics. This distance is computed with respect to a particular representation ϕ(·), a feature map function. This function can map the original data into a reproducing kernel Hilbert space (RKHS) endowed with a characteristic kernel *k*. The RKHS may be infinite dimensions that can transform non-separable data to linearly separable. The distance between the source domain with probability *p* and the target domain with probability *q* is defined as $d_{k}\left(p,q\right)$. The data distribution p=q iff $d_{k}^{2}\left(p,q\right)=0$. Then, the squared expression of MK-MMD distance [29] is denoted as Formula (4):

(4) $$d_{k}^{2}\left(p,q\right)\overset{\Delta }{\;=}{∥E_{p}\left[\phi (x_{S})\right]-E_{q}\left[\phi (x_{T})\right]∥}_{H_{k}}^{2}$$

where $H_{k}$ denotes the RKHS endowed with a characteristic kernel *k*.

Kernel technique, as Formula (5) shows, can be used to compute Formula (4), which can convert the computation of the inner product of the feature map ϕ(·) to computing the the kernel function k(·) instead.

(5) $$k\left(x_{S},x_{T}\right)=\langle \phi \left(x_{S}\right),\phi \left(x_{T}\right)\rangle$$

As mean embedding matching is sensitive to the kernel choices, MK-MMD uses multi-kernel *k* to provide better learning capability and alleviate the burden of designing specific kernels to handle diverse multivariate data. It provides more flexibility to capture different kernels and leads to a principled method for optimal kernel selection.

Multi-kernel K is defined as the convex combination of kernels $\left\{k_{u}\right\}$ as in Formula (6) [28]:

(6) $$K\overset{\Delta }{\;=}\left\{k=\sum_{u=1}^{m}B_{u}k_{u}:\sum_{u=1}^{m}B_{u}=1,\;B_{u}\geq 0,\;\forall u\right\}$$

where the constraints on coefficients $\left\{B_{u}\right\}$ make the derived multi-kernel *k* characteristic.

## 3. The Proposed Method

### 3.1. The Overview of the Proposed Approach

**Figure 2:**
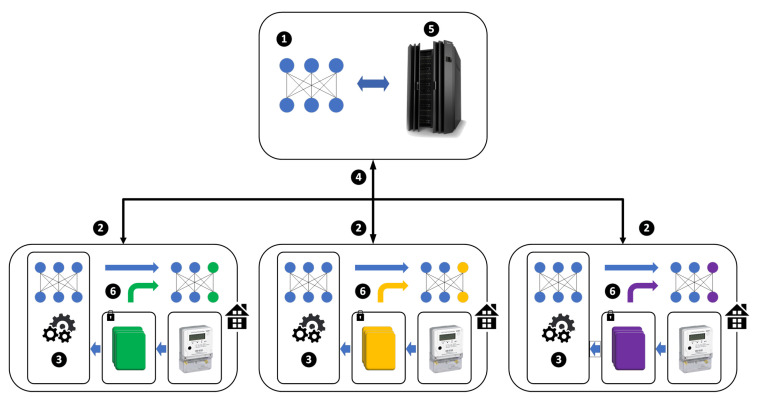
Overview of the deep federated adaptation. The top box is the master server while the 3 bottom boxes denotes 3 houses. Each house contains one computing device connected to the master server for processing the data. The data collected by the smart meter is locked and cannot be transmitted to the master server.

The overview of the proposed approach is shown in Figure 2. Without loss of generality, there are 3 households which need to be predicted, the number can be extended without too much work. Each household has a device for computing models and communicating with the master server. The approach mainly consists of 6 procedures as follows:

Step 1: The master server constructs the initial global model with public datasets.

Step 2: The master server distributes the global model to all users.

Step 3: The master server selects a fraction of users, then the selected devices train models with their local data.

Step 4: The selected devices upload models to the master server.

Step 5: The master server updates the global model by aggregating the uploaded models. Repeat Step 2 to Step 5 until the global model convergence.

Step6: Each device fine-tunes the convergent global model using user adaptation with local data.

### 3.2. Federated Learning Process

Deep neural networks are selected in the federated learning process since neural networks update models based on gradient descent. The federated learning process can get a pre-trained model for the latter user adaptation. Firstly, in the training process, the model is initialized on the master server with public datasets. The initial global model is denoted as $f_{G}$, then the learning objective function is defined as shown in Formula (7):

(7) $$\arg\min_{\Theta_{G}}L=\sum_{i=1}^{n}\ell \left(y_{i},f_{G}\left(x_{i}\right)\right)$$

where ℓ(·) denotes the loss for the neural network, the loss used in this paper is mean squared error (MSE) loss since load forecasting problem is a regression problem. ${\left\{x_{i},y_{i}\right\}}_{i=1}^{n}$ are samples from datasets, and Θ are the parameters learned.

After the initial global model is trained, the master server will distribute the model to all remote devices. Then, a subset of remote devices are chosen for training user model $f_{u}$ with local data. Let ${\left\{x_{i}^{u},y_{i}^{u}\right\}}_{i=1}^{n^{u}}$ denote samples from datasets *u*. Technically, the learning objective function for each user is denoted as Formula (8):

(8) $$\arg\min_{\Theta_{u}}L=\sum_{i=1}^{n^{u}}\ell \left(y_{i}^{u},f_{u}\left(x_{i}^{u}\right)\right)$$

Then, all the user models are uploaded to the master server for averaging based on the algorithm FedAVG [30], and the formulation of averaging is as Formula (9):

(9) $${f_{G}}^{\prime }\left(w\right)=\frac{1}{K}\sum_{k=1}^{n}f_{u_{k}}\left(w\right)$$

where *w* are parameters of the network and *K* is the number of devices in the chosen subset. Then, let $f_{G}=f_{G}^{\prime }$ on the master server, after adequate rounds of iterations, the updated server model $f_{G}$ has better performance on generalization ability. When devices of newly built houses are connected to the federated system, the master server can distribute the global model to help new devices take part in the next iteration, hence, federated learning can deal with cold start problems and is extensible. It is worth noting that the network is trained by the federated learning using data from different houses, which expands the training data and makes the model more robust, and has better generalization ability.

### 3.3. User Adaptation with Multiple Kernel Variant of Maximum Mean Discrepancies

Federated learning solves problems of data availability and privacy. However, another important problem is personalization. Even if the cloud model can be directly used, it still performs poorly on a particular house. The weights of this network have been pre-trained by the federated learning process, then the user adaptation process will fine-tune the pre-trained network. Since the network does not need to update all weights from scratch for new tasks, it costs less in computation and time, which is especially suitable for edge devices.

**Figure 3:**
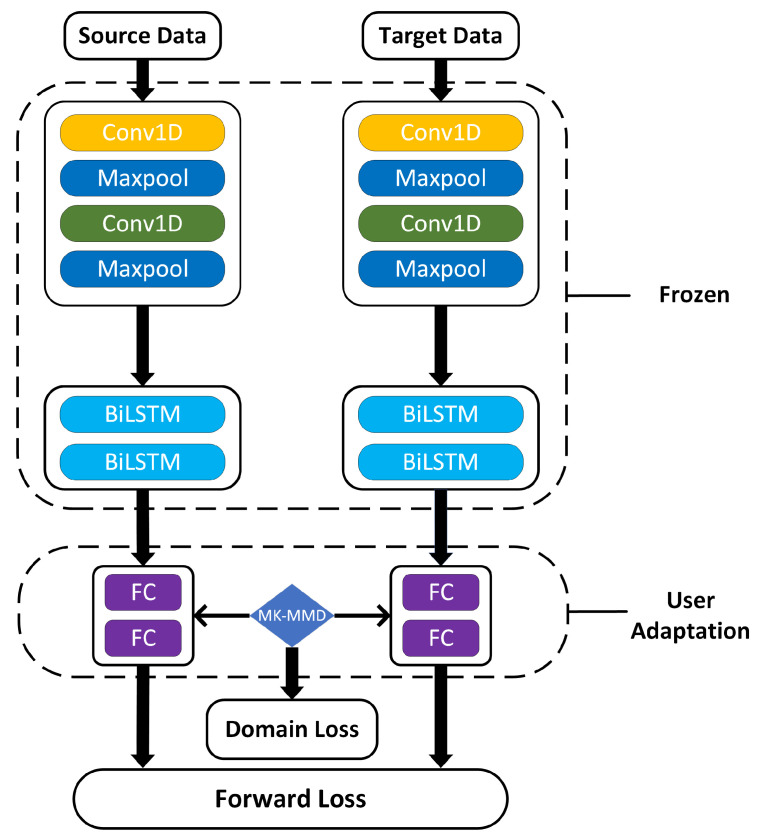
The architecture of proposed network, from top to bottom, consists of CNN layers, BiLSTM layers and fully connected layers.

Figure 3 shows the architecture of the proposed network. This is a classic hybrid model of convolutional neural network (CNN) and Bi-directional Long Short-Term Memory (BiLSTM), referred to as CNN-LSTM, more details can be found in [31]. This network is a two-stream architecture, thus the source data and the target data can be fed into the network simultaneously. Two streams of data go from the CNN layers to the Bi-LSTM layers and finally through the fully connected (FC) layers to compute the forward loss. We consider that sections of CNN can extract low-level features about a series of load values and BiLSTM aims at capturing sequential relationships.

To minimize domain discrepancy, domain loss is also introduced to optimize the network. MK-MMD is used to measure domain loss in which the source data is aligned with the target data for computing. Multi-kernel *k* is used to adapt to different feature domains and hidden representations of higher layers are embedded in a RKHS where the mean embeddings of distributions in different user data can be explicitly matched. The loss of MK-MMD is defined as shown in Formula (10) [23]:

(10) $$L_{MK-MMD}\left(X_{S},X_{T}\right)={∥\frac{1}{\left|X_{S}\right|}\sum_{x_{s}\in X_{S}}\phi \left(x_{s}\right)-\frac{1}{\left|X_{T}\right|}\sum_{x_{t}\in X_{T}}\phi (x_{t})∥}_{H}^{2}$$

where $x_{s}\in X_{S}$ denote source data points from source datasets and $x_{t}\in X_{T}$ denote target points from datasets of houses need to be adapted. Gaussian kernels are selected as the kernel function *k* in this paper since they can map features to infinite dimensions. We use a combination of Gaussian kernels by varying bandwidth γ with a multiplicative step size of $2^{1/2}$. The Gaussian kernel function with the bandwidth γ is defined, as shown in Formula (11):

(11) $$k\left(x_{s},x_{t}\right)=e^{-\frac{{∥x_{s}-x_{t}∥}^{2}}{\gamma }}$$

let η denote the trade-off parameter, then the total loss function of the network during user adaptation is computed by Formula (12):

(12) $$\arg\min_{\Theta_{u}}L_{u}=\sum_{i=1}^{n}\ell \left(y_{i},f_{u}\left(x_{i}\right)\right)+\sum_{i=1}^{n^{u}}\ell \left(y_{i}^{u},f_{u}\left(x_{i}^{u}\right)\right)+\eta L_{MK-MMD}\left(X_{s},X_{T}\right)$$

Since learned features transition from general to specific along the network with increasing domain discrepancies, the lower CNN and BiLSTM layers catch general features that can transfer from different houses. Hence, the parameters in the first dashed box in Figure 3 are frozen during user adaptation, whereas the weights of FC layers are updated by the total loss, as shown in Formula (12).

### 3.4. Learning Process and Summary

The learning procedures of DFA are summarized in Algorithm Section 3.4. Furthermore, we can consider the algorithm as a general process applied in STLF and separate the procedures into two sections. Section 1 of step 1 to step 8 is a federated learning process, while Section 2 of step 9 is for transfer learning. Other federated learning methods (e.g., vertically federated learning) can replace the horizontal federated learning method in Section 1 to deal with heterogeneous features from diverse organizations. Meanwhile, other effective transfer learning methods can also be embedded in Section 2 for better personalization. The neural network used in this framework can also be replaced according to the computing power of real-world devices or the features of datasets.

    **Algorithm** **1:** DFA: Deep Federated Adaptation        **Require:** Data from different houses $\left\{D_{1},D_{2},\cdots ,D_{N}\right\}$        **Ensure:** Adaptative forecasting models $f_{u}$          1: Build an initial global model $f_{G}$ with public datasets using (7)          2: **repeat**          3:     Distribute $f_{G}$ to all computing devices          4:     Use local data to train $f_{u}$ based on $f_{G}$ with (8)          5:     Devices upload $f_{u}$ to the model server          6:     Average models with (9)          7:     Update the global model $f_{G}={f_{G}}^{\prime }$          8: **until**
$\left|\Theta_{G}-{\Theta_{G}}^{*}\right|<\varepsilon$          9: Use (12) to optimize the convergent global model $f_{G}$, get adaptative models $f_{u}$

## 4. Experiments

### 4.1. Datasets Description and Pre-Processing

**Figure 4:**
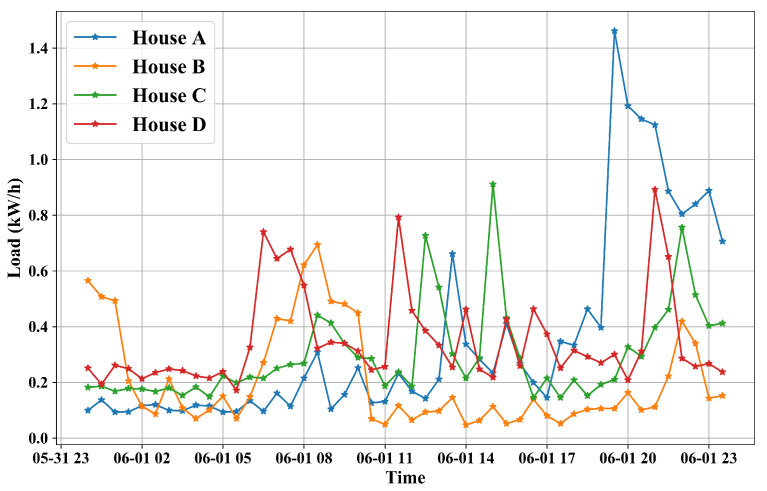
Load data of four houses for one day from the used datasets.

The experimental datasets are electricity consumption readings for a sample of 5567 London Households that took part in the UK Power Networks led Low Carbon London project between November 2011 and February 2014. Readings were taken at half-hourly intervals. The customers in the trial were recruited as a balanced sample representative of the Greater London population. The dataset contains electricity consumption, in kWh (per half hour), unique household identifier, date and time [32]. As an example, a period of records from 4 households are shown in Figure 4. It can be seen that the records are in different patterns which means a general model is not suitable for forecasting electricity consumption for a particular house. Meteorological variables recorded in London collected from Dark Sky API [33] are introduced to enrich our datasets. We merge electricity consumption datasets and meteorological datasets in terms of timestamps to generate a new feature table for each household.

Some discrete features (e.g., ’weekday’, ’icon’) should be encoded to embedding features. Then, feature normalization is implemented for all features with min–max normalization, as shown in Formula (13):

(13) x^ij=xij−xi,minxi,max−xi,min

where $x_{i}^{j}$ denotes the value for feature *i* at the time step *j*, $x_{i,min}$ and $x_{i,max}$ denote the maximal and minimal value for feature *i*, respectively. x^ij is the value for $x_{i}^{j}$ after normalization.

We consider the feature table as time-series data according to the timestamps, each row of the table denotes a record sampled at half-hourly intervals. We implement a sliding window with a look back at 24 records to forecast the next record. Hence, the proposed network can give a half-an-hour-later load value prediction, one training sample consists of features of 24 records and the value of the next electricity consumption. The input dimension is $\left|X\right|\times L$, where $\left|X\right|$ is the number of expected features in the merged table X, *L* denotes the width of the sliding window.

### 4.2. Implementation Information

The proposed network is composed of two convolutional layers, two pooling layers, two BiLSTM layers and two FC layers. The network adopts a convolution size of 1×17 and a kernel size of 3 for pooling layers. The proposed network is trained with the MSE loss and adopts stochastic gradient descent (SGD) with an initial learning rate of 0.01 and 0.9 momentum for optimization. Batch size is set to 32. The training process is early stopped within 10 epochs and the rate of dropout is set to 0.1 to prevent overfitting.

In the following experiments, cross validation and grid search are used to select the hyperparameters and the hyperparameters with the lowest average forecasting MAPE will be used. During the training process, we use 70% of the data for training while the rest 30% is for evaluation. All experiments are repeated five times to ensure reliability, implemented in Pytorch, and conducted on a single NVIDIA GeForce RTX 2080 GPU.

A single machine is used to simulate the federated learning process and we can set the number of user nodes $N_{nodes}$ according to the experimental requirements. Table 1 shows some symbol definitions of the experiments. Since a single machine is used to simulate the federated process, the training process is serial. However, this has no effect on comparing model forecasting accuracy and computation time between the federated architecture and the centralized architecture. Centralized learning means data are gathered from all devices to train a single model on the central server, which does not secure the privacy of users.

To evaluate the forecasting performance of DFA, four baseline models are used for comparison purposes. The following are simple introductions for these models.

1. LSTM network: the model is an artificial recurrent neural network (RNN) architecture with feedback connections used in the field of deep learning.
2. Double seasonal Holt–Winters (DSHW): DSHW is a kind of exponential smoothing method which can accommodate two seasonal patterns besides parts of trend and level.
3. Transformer: it is a deep learning model that adopts the mechanism of self-attention, differentially weighting the significance of each part of the input data.
4. Encoder–Decoder: the model encodes the input as embedding features which are decoded by the decoder, adopting a sequence-to-sequence architecture.

### 4.3. Model Evaluation Indexes

The mean absolute percentage error (MAPE) is used to evaluate forecasting accuracy. The evaluation equations are defined as shown in Formula (14):

(14) $$\mathrm{MAPE}=[\sum_{i=1}^{N}\left({\hat{y}}_{i}-y_{i}\right)/y_{i}]/N\times 100\%$$

where ${\hat{y}}_{i}$ is the forecast load consumption value, $y_{i}$ is the actual load consumption value and *N* is the total number of sampling points for evaluation.

To evaluate whether a particular model *m* has skill with respect to a baseline model *r* the MAE ratio, we use skill score, as shown in Formula (15):

(15) $$s=1-\frac{{\mathrm{MAE}}_{m}}{{\mathrm{MAE}}_{r}}$$

where MAE is the mean absolute error. MAE is calculated as shown in Formula (16):

(16) $$\mathrm{MAE}=[\sum_{i=1}^{N}\left|{\hat{y}}_{i}-y_{i}\right|]/N$$

### 4.4. Experimental Forecasting Performance

**Figure 5:**
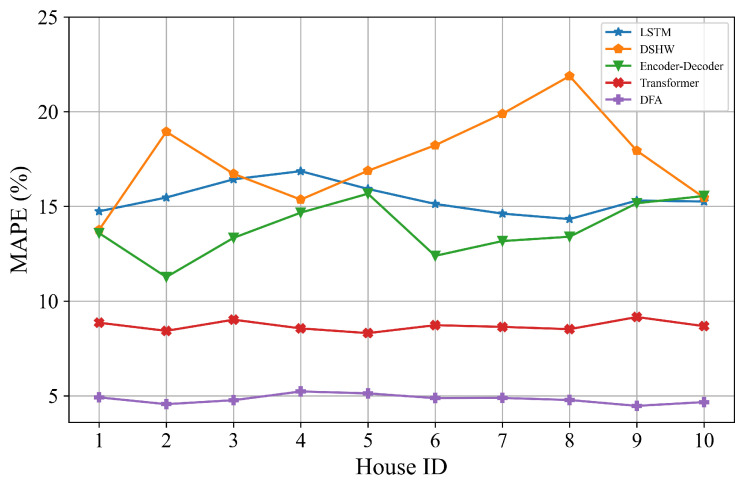
MAPE values of DFA and four baseline models for 10 houses.

The proposed DFA and four baseline models are evaluated on 10 randomly chosen target houses. For each target house, the load records from June 2012 to June 2013 are used as training data, and 720 load records in September 2013 for prediction to calculate MAPE values. DFA makes use of all datasets of ten houses in the federated process and leverages the datasets from the target house to operate user adaptation. Baseline models are trained with the data from the target house. Table 2 shows the MAPE values of DFA and baseline models for 10 houses. Figure 5 shows the MAPE values for direct observation.

**Figure 6:**
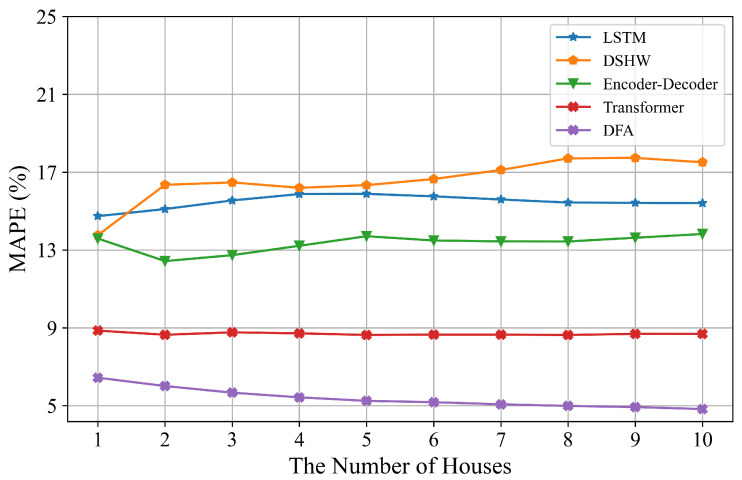
MAPE values of four baseline models and DFA with different numbers of houses connected to the federated system for 10 houses.

From Table 2, we can see that the proposed DFA consistently outperforms the baseline models for ten houses. On average, it shows 38.28%, 69.83%, 58.81% and 63.65% relative improvements over Transformer, DSHW, Encoder–Decoder and LSTM, respectively, based on skill scores. The performances of LSTM and Encoder–Decoder are similar to each other and worse than Transformer since the number of parameters is less compared to Transformer. Performances of DSHW fluctuate widely and are inferior to the other models based on deep learning. We believe this is due to the differences in the cyclical characteristics in different spans which are influenced by many uncertain factors in residential loads. In summary, DFA has the best performance. We conclude that one of the reasons for the remarkable superiorities is DFA uses all datasets from ten houses to learn a model in the federated architecture. Additionally, we calculate the curve of MAPE values by varying the number of houses as shown in Figure 6. It can be seen that MAPE values of DFA gradually decrease with the number of houses increasing whereas other models do not vary much. This means that the model will be more robust when more devices are connected to the systems in the reality. More discussions about the superiorities in forecasting performance can be found in Section 4.6.

To evaluate the persistence of DFA, we conduct day-ahead and week-ahead forecasting tasks of DFA and four baseline models on one house, the results are shown in Table 3. It can be observed that although the forecasting performance of DFA decreases as the period goes from one day to one week, DFA outperforms all baseline models no matter how long is the forecasting period. We attribute this decline to the fact that DFA uses a sliding window for training and forecasting: the value forecasted by DFA will be added to the end of the sliding window for the next forecasting. Forecasting errors are cumulative as the period grows.

### 4.5. Performance of Federated and Centralized Architecture

Table 4 shows the forecasting performance and computation time comparison of the federated and centralized architecture. CNN-LSTM, as shown in Figure 3, is chosen as the test model. The different number of records in Table 3 means how many records for each node are used to train the model. For federated learning, the training time can only be estimated, the training time can be estimated as shown in Formula (17):

(17) $$T_{training}={\overset{¯}{T}}_{round}\cdot N_{round}$$

where $T_{training}$ denotes the training time, ${\overset{¯}{T}}_{round}$ indicates average computation time for all devices involved in each round.

From Table 3, it can be seen that the forecasting performance of the federated architecture is superior to the centralized while making predictions for STLF in the conditions of the different numbers of local records with $N_{nodes}=10$. When $N_{nodes}$ and records increase, the federated architecture can make use of more data to train the model, the forecasting performance will improve.

The federated architecture outperforms the centralized architecture on computation time comprehensively, with great advantage. As the federated architecture leverages devices involved in each round for training at the same time. It can be seen that the computation time fluctuates only slightly when $N_{nodes}$ increases. This is because in each round, each device only processes the local data simultaneously and does not care about data from other devices in the system. Meanwhile, it also can be observed that the computation time rises at a lower rate than the centralized method as the number of records increases because for the centralized architecture, the incremental data of each device should be collected for training, the increment of data is decided by $N_{nodes}$.

**Figure 7:**
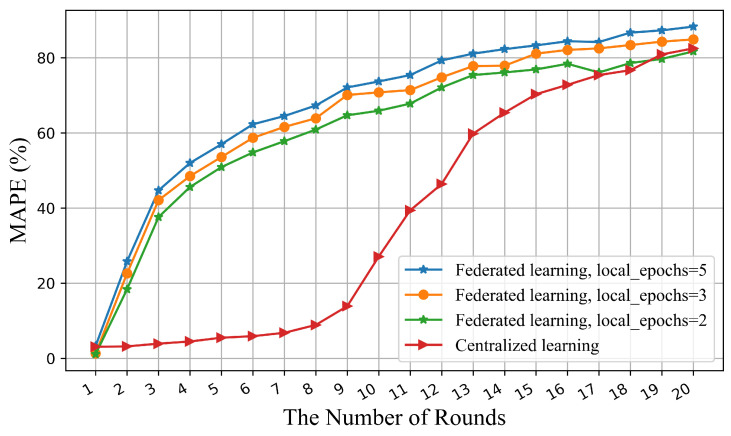
Correlation between accuracy and number of rounds for the federated and centralized architecture.

Figure 7 shows the correlation between accuracy and the number of rounds for these two architectures. Federated learning shows a higher rate rise at the beginning of the iterations while the centralized accuracy rises slowly because multiple devices compute simultaneously in one round of iterations of federation learning. As can be seen from the trend of the curves, the federated architecture uses fewer iterations to achieve a satisfactory accuracy and achieve the state of convergence, which is also reflected in the shorter computation time in Table 3.

Now, we analyze the communication overhead of these two architectures. For the federated architecture, the calculation of communication overhead is defined in Formula (18):

(18) $$Trans_{Fed}=2N_{round}\cdot N_{nodes}\cdot S_{model}$$

meanwhile, the communication overhead of the centralized architecture is defined as shown in Formula (19):

(19) $$Trans_{Cen}=N_{nodes}\cdot S_{data}$$

From the aforementioned formulas, the communication complexity of the federated architecture is $O(N_{round}\cdot N_{nodes}\cdot S_{model})$ and the centralized architecture is $O(N_{nodes}\cdot S_{data})$. Since $N_{nodes}$ is presented in both equations, it can be reduced. Therefore, the complexity is $O(N_{round}\cdot S_{model})$ and $O(S_{data})$ respectively. When the $S_{data}$ is much larger than $N_{nodes}\cdot S_{data}$, the federated architecture has less communication burden than the centralized, which is common in practical applications. We can easily infer that the computation time will increase in the reality since the incremental communication overhead. In a summary, DFA is scalable with increasing data and has lower computation time and communication bandwidth requirements.

### 4.6. Ablation and Extensibility Experiments

To validate the superiority of DFA, we conduct the ablation and extensibility experiments based on datasets of five houses, other experiment settings are consistent with Section 4.4.

**Figure 8:**
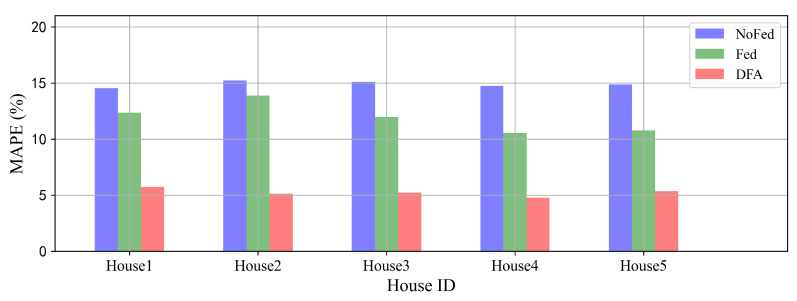
Ablation experiments of the federated architecture and MK-MMD optimization on 5 houses.

We use Fed to denote the DFA without MK-MMD optimization which is a CNN-LSTM network trained by the federated architecture. NoFed denotes the CNN-LSTM model trained by the centralized architecture with data only from the target house. We can see from Figure 8 that Fed achieves a better performance than NoFed on each target house. This indicates that each target house benefits from the federated architecture which makes it possible to leverage datasets from other houses, ensuring privacy simultaneously. It also can be seen that DFA has remarkable improvements in performance compared with Fed. We conclude that the transfer learning method can successfully conduct knowledge transfer from the federated model to the target houses to improve forecasting performance.

**Figure 9:**
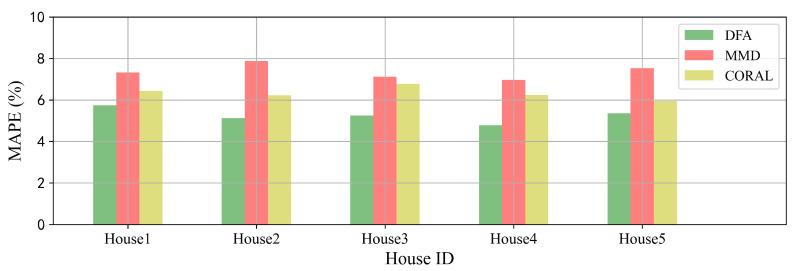
Extensibility experiments with alternative transfer learning methods on 5 houses.

Furthermore, we extend DFA to different versions in which the part of MK-MMD is modified by the alternative transfer learning methods. Maximum mean discrepancy (MMD) is the single kernel version of MK-MMD. CORAL [34] is one of transfer learning methods that use the covariance matrices of the source and target features to compute the domain loss. It can be seen from Figure 9 that DFA can achieve satisfying performances on forecasting with different transfer learning methods. The results indicate that DFA is extensible with other transfer learning algorithms according to the real applications.

## 5. Conclusions

In this paper, we propose a federated transfer learning approach for residential STLF. This approach addresses data availability and privacy by using a federated architecture. We implement a transfer learning method, multiple kernel variant of maximum mean discrepancies, to adapt to the non-IID data among different houses. The experimental results show that DFA shows a huge improvement in forecasting performance over other models. We also evaluate the federated architecture DFA used; it shows that the architecture is superior to the centralized architecture in computation time and has a small burden on communication. In the future, it would be promised for subsequent studies to adopt the state-of-the-art federated and transfer learning algorithms to achieve better forecasting performance with the framework of DFA.

## Figures and Tables

**Table 1:** Symbol definitions of the experiments.

Name	Description
$N_{nodes}$	Number of computing nodes
$N_{round}$	Number of iterations
$S_{data}$	Size of the trained data
$S_{model}$	Size of the model

**Table 2:** MAPE values of DFA and baseline models for 10 houses.

DataSets	DFA	Transformer	DSHW	Encoder–Decoder	LSTM
House 1	4.92%	8.86%	13.76%	13.59%	14.74%
House 2	4.56%	8.43%	18.94%	11.27%	15.47%
House 3	4.77%	9.02%	16.72%	13.34%	16.43%
House 4	5.23%	8.56%	15.36%	14.67%	16.86%
House 5	5.13%	8.31%	16.88%	15.67%	15.92%
House 6	4.88%	8.73%	18.23%	12.39%	15.13%
House 7	4.89%	8.64%	19.89%	13.17%	14.62%
House 8	4.78%	8.52%	21.88%	13.40%	14.33%
House 9	4.47%	9.16%	17.94%	15.17%	15.31%
House 10	4.67%	8.68%	15.46%	15.55%	15.26%
Average	4.83%	8.69%	17.51%	13.82%	15.41%

**Table 3:** Forcasting MAPE values of DFA and baseline models for day-ahead and week-ahead.

Model	Day 3	Day 11	Day 15–Day 21
**DFA**	6.43%	6.17 %	8.83 %
**DSHW**	16.85%	17.23%	17.61%
**Transformer**	8.77%	9.22 %	10.24%
**Encoder–Decoder**	13.64%	14.56 %	16.79%
**LSTM**	18.23%	17.64 %	21.54%

**Table 4:** MAPE values and computation time of the federated and centralized architecture.

Approach	Forecasting Performance			Computation Time (s)		
Number of Local Records	5000	8000	15,000	5000	8000	15,000
Federated ($N_{nodes}=1$)	13.82%	13.42%	13.38%	5.26	5.58	5.73
Federated ($N_{nodes}=4$)	12.83%	12.33%	11.89%	5.38	5.73	5.84
Federated ($N_{nodes}=7$)	11.57%	11.35%	10.87%	5.23	5.46	5.91
Federated ($N_{nodes}=10$)	10.24%	10.13%	9.83%	5.47	5.62	5.88
Centralized ($N_{nodes}=10$)	12.13%	12.24%	12.07%	8.32	11.74	25.63
